# Genotypic and Phenotypic Characterization of *Treponema phagedenis* from Bovine Digital Dermatitis

**DOI:** 10.3390/microorganisms8101520

**Published:** 2020-10-02

**Authors:** Hector M. Espiritu, Lovelia L. Mamuad, Su-jeong Jin, Seon-ho Kim, Seok-won Kwon, Sang-suk Lee, Sang-myeong Lee, Yong-il Cho

**Affiliations:** 1 Sunchon National University, Suncheon, Jeonnam 57922, Korea; 1193001@s.scnu.ac.kr (H.M.E.); loveliamamuad2306@gmail.com (L.L.M.); herodias@scnu.ac.kr (S.-j.J.); mhs0425@daum.net (S.-h.K.); rumen@scnu.ac.kr (S.s.-L.); 2 Woosarang Animal Hospital, Yongin, Gyeonggi 17026, Korea; gastra00@naver.com; 3 Jeonbuk National University, Iksan, Jeonbuk 54596, Korea; leesangm@jbnu.ac.kr

**Keywords:** lameness, bovine digital dermatitis, *Treponema phagedenis*

## Abstract

This study aimed to isolate and characterize *Treponema* spp. from bovine digital dermatitis (BDD)-infected dairy cattle. Seven isolates were characterized in this study. Isolates exhibited slow growth, and colonies penetrated the agar and exhibited weak β-hemolysis. Round bodies were observed in old and antibiotic-treated cultures. Cells ranged from 9–12 µm in length, 0.2–2.5 µm in width, and were moderately spiraled. The 16S rRNA analysis revealed the isolates as *Treponema phagedenis* with >99% sequence homology. Isolates had alkaline phosphatase, acid phosphatase, β-galactosidase, N-acetyl-β-glucosaminidase, esterase (C4), esterase lipase (C8), naphthol-AS-BI-phosphohydrolase, and β-glucuronidase activities. Low concentrations of ampicillin, erythromycin, and tetracycline were required to inhibit the growth of isolates. Formic, acetic, and butyric acids were produced, while propionic acid was significantly utilized, indicating its essentiality for treponemal growth. The isolates shared the same characteristics and, therefore, were considered as a single strain. Isolate HNL4 was deposited as a representative isolate (*Treponema phagedenis* KS1). The average nucleotide identity of strain KS1 showed a small difference with the human strain (99.14%) compared with bovine strain (99.72%). This study was the first to isolate and characterize *Treponema phagedenis* from BDD in Korea and, hence, it delivered pathogenicity-related insights and provided valuable information that can be used for the management of BDD.

## 1. Introduction

Bovine digital dermatitis (BDD) is an important infectious foot disease causing lameness in cattle [1]. Characterized by ulcerative dermatitis, it later forms a hyperkeratotic papillomatous lesion [2] which is highly painful and sensitive when touched or manipulated, and it often leads to bleeding [3]. Its main clinical sign is lameness, and infected animals are usually observed to walk on their toes or to stand on a hind leg with frequent shifting of weight from foot to foot [4]. Serious issues associated with BDD lead to increasing concerns about the reduced welfare of the animal as well as significant economic losses due to the facts of reduced milk yield, premature culling, and poor reproductive performance [5].

For almost half a decade since the initial report of BDD in Italy [6], BDD has been subsequently reported in various parts of the world, reaching an endemic state in many countries [7]. Some studies on BDD have focused on determining its etiology and pathogenesis with the expectation of developing an efficient treatment [8]. Extensive investigations have revealed that diverse *Treponema* spp. are consistently described in BDD lesions [7,9,10], suggesting it as polytreponemal [4,11]. Thus far, three phylogroups—*T. pedis*, *T. medium*, and *T. phagedenis*—have been reported as being the most prevalent and abundant [7,12]. Among these, *T. phagedenis* is considered a key agent in the pathogenesis due to the fact of its presence in the interface with healthy tissues [13]. 

Despite being globally widespread, unexpectedly, there are limited reports on BDD and BDD-associated treponemes. The fastidiousness of *Treponema* makes it difficult to culture in vitro which has limited the success of studies aimed at determining the pathogenic mechanisms of these bacteria. Only a few researchers have successfully isolated and characterized BDD-associated treponemes [1,2,11,14,15,16,17,18]. Recently, an investigation on BDD in Korea investigated the microbiome diversity and the dominant pathogens in lesions [9]. This study aimed to isolate and characterize *Treponema* spp. from BDD lesion on dairy cattle by phenotypic and genotypic approaches, as well as determining its short-chain fatty acid requirement and minimum inhibitory concentration.

## 2. Materials and Methods 

### 2.1. Collection, Isolation, Growth, and Microscopy

Samples were collected from active BDD lesions of Holstein–Friesian cows from dairy cattle farms in Korea by a professional veterinarian. After thorough cleaning of the foot, lidocaine (2%) was injected around the lesion and a 5 mm punch biopsy was taken and thoroughly washed with phosphate-buffered saline (PBS) with pH 7.4. Afterward, samples were ground and inoculated to oral *Treponema* enrichment broth (OTEB) (Anaerobe System, Morgan Hill, CA, USA) supplemented with 10% fetal bovine serum (FBS), rifampicin (10 µg/mL), and enrofloxacin (10 µg/mL), and then incubated anaerobically at 37 °C for 48–72 h. The OTEB culture was subcultured onto fastidious anaerobe agar (FAA) supplemented with 5% defibrinated sheep blood, 10% FBS, and antibiotics. Plates were incubated anaerobically at 37 °C and were observed every seven days until growth was observed. A single colony from the FAA was cultured in OTEB with supplementation as above mentioned to achieve isolate purity.

To determine the growth rate, a hundred microliters of 5 McFarland standardized-bacterial suspension were inoculated in OTEB tubes supplemented as above. The growth rate was analyzed by measuring the optical density at 630 nm at 0, 3, 7, and 14 day incubation. 

Field emission scanning electron microscopy (FE-SEM) was conducted at Cheonnam National University, Yeosu Campus. Bacterial cells obtained from 7 day broth culture washed with PBS were pre-fixed with 2.5% glutaraldehyde for 4 h at 4 °C. Then, post-fixation was performed using 1% osmium tetroxide at 4 °C for 2 h (dark condition). Samples were soak-washed thoroughly with PBS, then gradient-washed with ethanol (30% to 99%), air-drying each wash. Platinum coating was applied at 10 mA for 1 min. Imaging was done under a Sigma 500 FE-SEM (Zeiss, Oberkochen, Germany).

### 2.2. Molecular Characterization

The 16S rRNA gene was amplified using the universal primers 27F and 1492R. The flagellin gene was amplified using primers targeting a 750 bp region for flagellar protein (*flaB2*) [19]. Amplicons were purified and were submitted to Macrogen Inc. (Seoul, Republic of Korea) for sequencing. Assembled sequences of 16S rRNA and the partial region of *flaB2* gene were identified using BLAST. Phylogenies of each gene were constructed using the maximum likelihood (ML) method. 

The whole genome sequence of the representative isolate (HNL4—*T. phagedenis* KS1) was obtained by reference-guided assembly using Bowtie v.2. Using the Illumina HiseqXTen platform (Illumina Inc., San Diego, CA, USA), the sequenced paired-end reads were filtered and assembled to the reference genome of *T. phagedenis* B43.1 (NZ_CP042818). The average nucleotide identity (ANI) of *T. phagedenis* genome and construction of phylogenomic tree by the unweighted pair group method with the arithmetic mean (UPGMA) were conducted using the OrthoANI Tool [20]. The genome of the strain KS1 described in this study was the reference-guided version with GenBank, EMBL, and DDBJ accession CP054692.

### 2.3. API^®^ ZYM, Minimum Inhibitory Concentration (MIC), and Short Chain Fatty Acids (SCFA)

The biochemical activity was assessed using API^®^ ZYM test system (bioMérieux Inc., Marcy-l’Étoile, France) following the manufacturer’s standard procedure. Test incubation was done anaerobically at 37 °C.

The minimum inhibitory concentration (MIC) was measured by broth micro-dilution method following the previous protocol for the assay and control validation [21,22]. The panel consisted of six antimicrobial agents: ampicillin, enrofloxacin, erythromycin, kanamycin, rifampicin, and tetracycline, diluted in two-folds from 0.5 µg/mL to 256 µg/mL.

Sample preparation and measurement of short-chain fatty acids (SCFAs) were done following the published method for volatile fatty acid quantification using high-performance liquid chromatography (HPLC) [23]. The standardized bacterial suspension (5 McFarland) was inoculated in OTEB. A milliliter sample was obtained from each culture at 3, 7, and 14 day incubation and stored at –80 °C until HPLC measurement. Comparison of SCFA quantity among isolates and times was done using ANOVA with the general linear model. Significant differences between isolates and times were compared using Duncan’s multiple range test. All data were analyzed using SAS v.9.3 (SAS Institute Inc., Cary, NC, USA).

## 3. Results

### 3.1. Isolation, Growth, and General Characteristics

**Figure 1:**
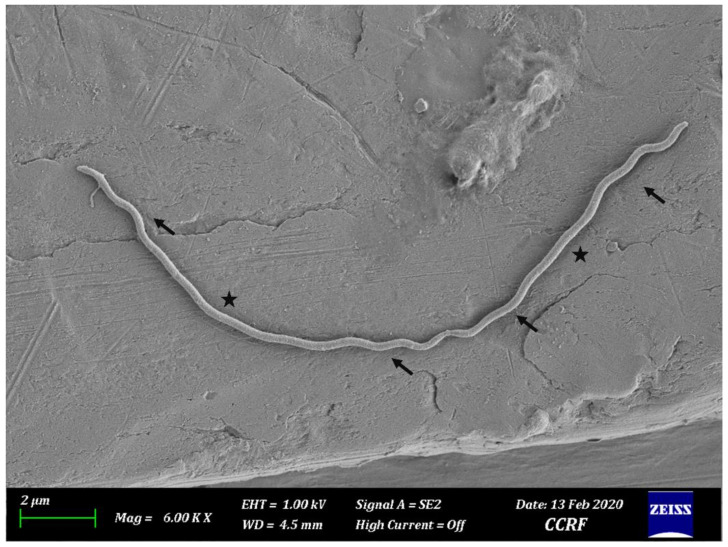
*Treponema phagedenis* cells under FE-SEM displaying moderate helical coils on both ends of the cells (arrow) with a semi-coiled region in the middle portion of the cells (star) at 6000× magnification.

**Figure 2:**
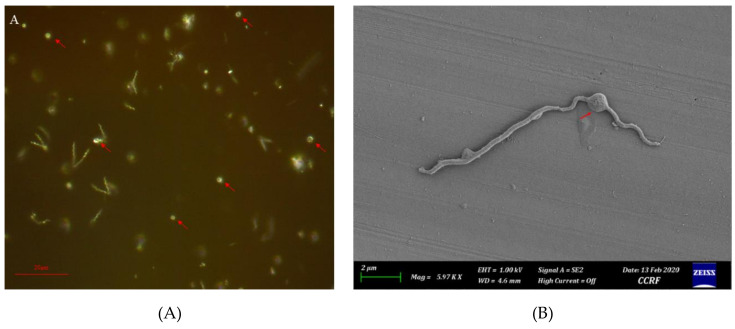
Round bodies in Oral *Treponema* Enrichment Broth (OTEB) culture with antibiotics (i.e., rifampicin and enrofloxacin) under (**A**) dark field microscope (400×, 21 day) and (**B**) FE-SEM (~6000×, 7-day).

**Figure 3:**
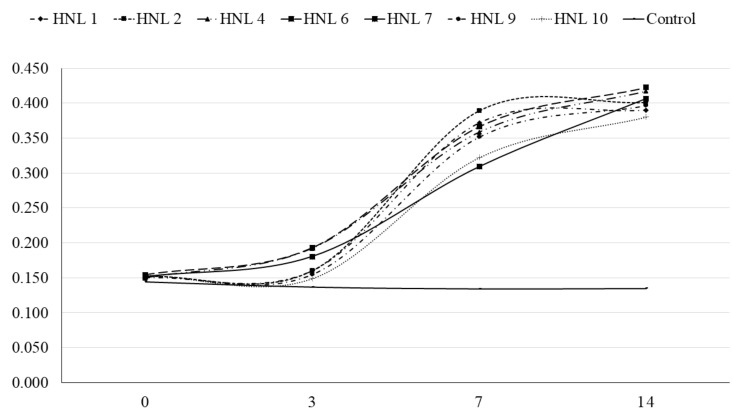
Optical density (630 nm) of *T. phagedenis* isolates OTEB culture from 0 to 14 days of incubation at 37 °C.

Seven bacterial isolates (HNL 1, 2, 4, 6, 7, 9, and 10) were purified. Streaks on FAA plates produced round opaque colonies ranging from 1 to 2 mm in diameter which penetrated beneath the agar surface, exhibiting weak β-hemolysis after 7–14 days of incubation. The FE-SEM imaging revealed varying cell sizes ranging from 9 µm to 12 µm in length and 0.2–0.25 µm in thickness (Figure 1). Morphology of bacterial cells can be characterized as generally less helical, having two main regions, a moderately helical region on both ends of the cell, and a semi-coiled form in the middle portion of the cell (Figure 1). Atypical forms or round bodies were also persistently observed in old and/or antibiotic-treated broth cultures under dark field and FE-SEM (Figure 2). The measured OD_630_ indicated notably slow growth. All isolates presented a significant increasing linear trend in growth throughout the observation periods (Figure 3).

### 3.2. Molecular Characteristics

**Figure 4:**
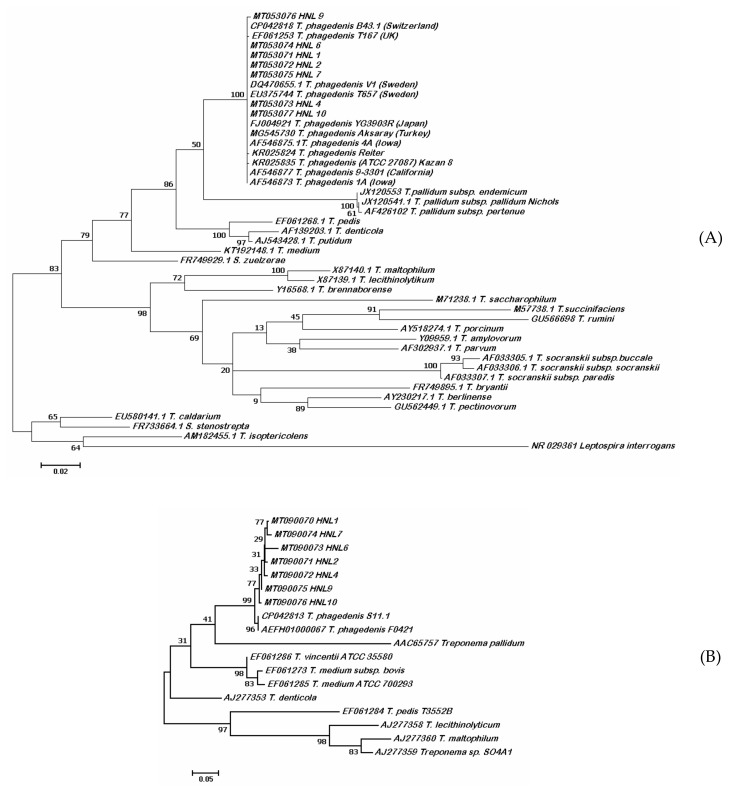
Maximum likelihood trees (**A**) 16S rRNA and (**B**) *flaB2* genes. Korean isolates clustered with other *T. phagedenis* isolates.

**Figure 5:**
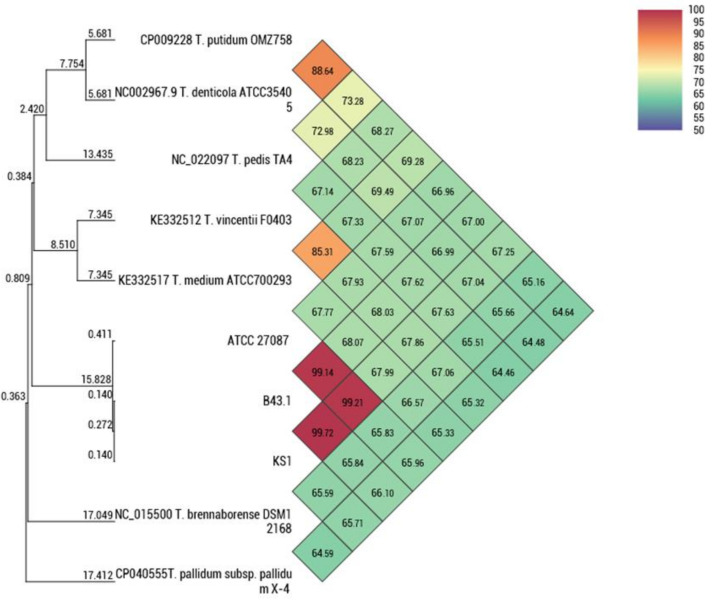
The unweighed paired group method with arithmetic mean (UPGMA) tree and heatmap based on average nucleotide identity (ANI) between *T. phagedenis* strain KS1 and other *Treponema* spp.

The BLAST search of the obtained ~1450 bp 16S rRNA sequences showed >99% identity with *T. phagedenis*. The constructed phylogeny inferred by maximum likelihood method based on the Tamura–Nei model showed the evolutionary relationships of the isolates from 21 *Treponema* species clustering with the *T. phagedenis* group. *Leptospira interrogans* was used as the outgroup (Figure 4A). The sequences were deposited in GenBank with accession numbers MT053071-77. Analysis of a 577 bp polymorphic sequence from the partial region of *flaB2* further inferred the identity of isolates using the ML method based on the Kimura-2 parameter model. The constructed tree of the *flaB2* showed that the isolates clustered with *T. phagedenis* (Figure 4B). Sequence data were deposited to GenBank with accession numbers MT090070-76. Isolate HNL 4 was selected as a representative strain and was deposited in the Korean Collection for Culture Types (KCTC, Jeongeup, Republic of Korea) as *Treponema phagedenis* KS1 with accession number KCTC14157BP designated by the International Depositary Authority. The average nucleotide identity (ANI) of *T. phagedenis* KS1 genome compared with bovine strain B43.1 was 99.72%, while it was 99.24% for the human strain (Figure 5). The phylogenomic relationship of strain KS1 with other *T. phagedenis* strains and with other *Treponema* spp. is presented as unweighed paired group method with arithmetic mean (UPGMA) tree (Figure 5).

### 3.3. API^®^ ZYM, Minimum Inhibitory Concentration (MIC), Short-Chain Fatty Acid (SCFA) Quantification

The biochemical characteristics of the isolates were assessed by API^®^ ZYM (Table 1). All seven isolates shared the same reaction intensities on the tested substrates. Strong enzyme activity was observed for alkaline phosphatase, acid phosphatase, β-galactosidase, and N-acetyl-β-glucosaminidase, while moderate reactions were observed for esterase (C4), esterase lipase (C8), naphthol-AS-BI-phosphohydrolase, and β-glucuronidase. *Treponema phagedenis* isolates from Iowa, USA, and Sweden shared similar enzyme activity levels with the Korean isolates.

The result of the broth micro-dilution assay is summarized in Table 2. All isolates showed the same response towards the antimicrobial agents. Ampicillin showed the lowest inhibitory concentration (<0.5 µg/mL), followed by erythromycin (<1 µg/mL) and tetracycline (2 µg/mL). Higher concentrations were required to inhibit the growth of the isolates for enrofloxacin, kanamycin, and rifampicin (128 µg/mL).

The result of the broth micro-dilution assay is summarized in Table 2. All isolates showed the same response towards the antimicrobial agents. Ampicillin showed the lowest inhibitory concentration (<0.5 µg/mL), followed by erythromycin (<1 µg/mL) and tetracycline (2 µg/mL). Higher concentrations were required to inhibit the growth of the isolates for enrofloxacin, kanamycin, and rifampicin (128 µg/mL).

The quantities of SCFA’s produced/utilized by the isolates are presented in Table 3. Formic acid was initially undetected, but a significant production was recorded on the 7th day of incubation. Isolate HNL 6 exhibited the highest level of formic acid production at that time. Regardless, only HNL 2 showed a significant linear trend for formic acid production. The isolates also showed significant production of acetic acid based on linear trend analysis at different times. Isolate HNL 7 consistently showed the highest acetate production level on the 3rd, 7th, and 14th days of incubation. All isolates exhibited butyric acid production, but only HNL 4 presented a significantly linear increase. Among the SCFA’s measured, only propionic acid displayed a significantly decreasing trend over time with total utilization after 14 days of incubation in all isolates except HNL 7, which showed a significant increase on day 14. This result indicates that propionic acid could potentially be an essential SCFA for the growth of *T. phagedenis*.

## 4. Discussion

This study reports the characterization of the first successfully isolated *Treponema* spp. in Korea from dairy cattle with bovine digital dermatitis. Difficulties in culturing BDD-associated treponemes, due to the fact of their fastidiousness has restricted the number of isolates and the recovery of treponemes collected from lesion samples. In *Treponema*-associated studies, concerns regarding the difficulty of isolating a pure culture has been emphasized [14,24]. Extensive cleaning was crucial in the elimination of contaminants, and the increased antibiotic concentrations allowed treponemal growth while inhibiting others’ growth. From several tissue samples, only one farm sample produced *Treponema* spp.; thus, this study focused on the characterization of these isolates. All seven isolates were identified molecularly as *T. phagedenis*. On a recent study on BDD in Korea, *T. phagedenis* was detected on 86.2% of the lesions by specific PCR detection, which indicated its high prevalence in the country. However, the metagenomics data showed 0% relative abundance on all samples subjected to the analysis [9].

Agar penetration and weak β-hemolysis on FAA plates were notable cultural characteristics. The morphological characteristics of the isolates observed under FE-SEM were similar to those previously described to be generally straighter morphologically [1]. The periplasmic flagellum (PF), one of the distinguishing features of treponemes, was not observable under FE-SEM. Round bodies, also known as atypical form, L-form, or cysts were also observed in old and antibiotic-treated cultures. Cultural and phenotypic characteristics could be used for the presumptive classification and determination of putative virulence factors. The agar penetration observed conveys the capability of this bacterium to penetrate through a solid medium, as observed in other chemotactic motile bacteria [25]. A study on *Treponema denticola*, a spirochete involved in human periodontitis, suggested that motility and chemotaxis are major virulence-related factors in its tissue penetration by disrupting oral epithelial tissues in-vitro [26]. *T. phagedenis* has been reported to invade deep within the epidermis and penetrate the stratum spinosum [17]. Hemolysis, another characteristic observed, is considered as a virulence factor by regulating the iron level by lysing the erythrocytes at infection sites in their immediate environment to increase the level of available iron [27]. *Treponema denticola* displayed an iron acquisition mechanism that involved binding a 47 kDa outer membrane sheath with hemin, an important iron source derived from hemoglobin [28]. Varying degrees of hemolysis by *T. phagedenis* from BDD have been reported [1,2,14,16], but this property still requires further investigation since BDD often involves a bleeding response. Furthermore, round bodies have been observed in other *Spirochetes*, especially on human pathogens, *Borrelia burgdorferi,* and *Treponema pallidum* subsp. *pallidum* which causes Lyme disease and syphilis, respectively. Round bodies are stress-induced spirochete which persists during starvation and are at risk for desiccation and can later revert to its spiral form when introduced to favorable conditions. Round body formation is a neglected defense mechanism of spirochetes [29]. Understanding its survivability during treatment could help manage better treatment strategies.

Molecular identification using the 16S rRNA and the *flaB2* gene revealed the evolutionary relationship of *T. phagedenis* Korean isolates from other *Treponema* species. The phylogenetic trees constructed using these genes confirmed the identity of the isolates. All isolates had >99% sequence homology for 16S rRNA and are therefore similar to each other and *T. phagedenis* isolates from other countries. The *flaB2* gene, on the other hand, is considered to be a putative virulence gene [30] and previous reports have shown that it is a valuable marker in the phylogenetic analysis and genotyping of treponemes [30,31]. The average nucleotide identity between *T. phagedenis* KS1 and other strains and *Treponema* spp. revealed the relationship. The small difference of ANI values between the human and bovine strain could imply host adaptation as previously described [1].

The enzyme profile of the isolates shared a common degree of substrate reactivity with isolates from Iowa (4A) and Sweden (V1). Other studies that evaluated the enzyme activities of *T. phagedenis* showed varying profiles. Although there may be variations in substrate reactivity among strains from differences in geographical locations, test conditions, and subjective interpretations, this can lead to mismatching results and interpretation.

The susceptibility of antimicrobials to the isolates showed the efficacy of ampicillin, erythromycin, and tetracycline to inhibit the growth of *T. phagedenis* at low concentrations. The result of the assay is in agreement with the Japan isolates [2], except for enrofloxacin which required higher concentration (4–16 µg/mL). It is expected though for rifampicin and enrofloxacin to require higher concentration because these are commonly used agents for selective culture due to the natural resistance of treponemes towards these agents as also applied in this study. Although, until now, there are no standard breakpoints to assess the resistance of *T. phagedenis* against antimicrobial agents, the data provided would be helpful to strategize treatment since *T. phagedenis* is a prevalent spirochete in BDD.

The measured SCFA showed that formic, acetic, and butyric acids were produced, while propionic acid was utilized after 14 days. Based on Bergey’s Manual of Systematic Bacteriology [32], there are no reports of SCFA production or utilization linked with *T. phagedenis*. They also added that there was no growth in SCFA-supplemented culture. *Treponema phagedenis* produces SCFAs and alcohol as metabolic end products of fermentation [33]. Furthermore, acetic and N-butyric acids are major fermentation end products of *T. phagedenis* Reiter, while propionic acid is often not common and/or not always present [34]. These SCFAs were also described to be not required for the growth of the Reiter strain of *T. phagedenis* [34]. In a previous report, *T. phagedenis* 4A (Iowa) from BDD produced formic, acetic, and butyric acids [18]. Unfortunately, quantitative data were not presented. The quantitative results for formic, acetic and butyric acids obtained in this study agree with previous reports. However, the result in this study shows complete utilization of propionic acid after 14 days of incubation, opposing those previously published, and indicating that *T. phagedenis* requires propionic acid for growth. Also, *Treponema* phylotypes have varying essential growth requirements, such as sugars and fatty acids. Production of SCFAs during infection may allow other *Treponema* phylotypes to utilize these metabolic end products. Therefore, extensive investigation of the production and utilization of fatty acids and other nutritional requirements by BDD-associated treponemes is required to develop a deeper understanding of the metabolic relationship among these microbes.

The *T. phagedenis* isolates in this study shared similar characteristics, indicating that they are a single strain. Cultural, morphological, biochemical, molecular, and genomic characteristics, as well as susceptibility to antimicrobials, and short-chain fatty acid requirement of *T. phagedenis* isolates from other countries indicates that strains could still vary phenotypically and genotypically based on culture characteristics, geographical location, and host adaptation. This study is the first successful isolation and characterization of *Treponema phagedenis* isolated from BDD in Korea and, hence, delivered pathogenicity-related insights and provided valuable information that can be used for the management and treatment of BDD. 

## Figures and Tables

**Table 1:** Enzyme activities of isolated *Treponema phagedenis* from bovine digital dermatitis lesions in South Korea as determined by API^®^ ZYM.

Isolate	Enzyme Activity																		
	1	2	3	4	5	6	7	8	9	10	11	12	13	14	15	16	17	18	19
Korean isolates ^*^	S	W	W	-	-	-	-	-	-	S	W	-	S	W	-	-	S	-	-
4a (Iowa, USA) ^c^	S	W	W	-	-	-	-	-	-	S	W	-	S	W	-	-	S	-	-
V1 (Sweden) ^b^	S	W	W	-	-	-	-	-	-	S	W	-	S	W	-	-	S	-	-
1452 (Iowa, USA) ^c^	S	W	W	-	-	-	-	-	-	S	W	-	S	S	W	-	W	-	W
Iowa isolates ^a^	+	+	+	-	-	-	-	-	-	+	+	-	+	-	-	-	+	-	-
Japan isolates ^d^	+	+	+	-	-	-	-	-	-	+	-	-	+	+	-	-	+	-	+
Turkey Group II ^e^	+	+	+	-	+	-	-	-	-	+	+	-	+	+	-	-	+	-	-
B43.1 (Switzerland) ^f^	+	+	-	-	-	-	-	-	-	+	+	-	+	-	-	-	+	-	-
Kazan ^a^	+	+	+	-	+	-	-	-	-	+	+	-	+	-	-	-	+	-	-

* Isolates in this study, ^a^ [18]; ^b^ [16]; ^c^ [15]; ^d^ [2]; ^e^ [14]; ^f^ [1]. (1) alkaline phosphatase, (2) C4 esterase, (3) C8 esterase lipase, (4) C14 lipase, (5) leucine arylamidase, (6) valine arylamidase, (7) cystine arylamidase, (8) trypsin, (9) α-chymotrypsin, (10) acid phosphatase, (11) Naphtol-AS-BI-phosphohydrolase, (12) α-galactosidase, (13) β-galactosidase, (14) β-glucuronidase, (15) α-glucosidase, (16) β-glucosidase, (17) N-acetyl β-glucosaminidase, (18) α-mannosidase, (19) α-fucosidase. (S) Strong positive reaction; (W) Weak positive reaction; (+) Positive reaction; (–) Negative reaction.

**Table 2:** The minimum inhibitory concentration of six antimicrobial agents to *T. phagedenis* isolates.

Isolate	MIC (µg/mL)					
	Ampicillin	Enrofloxacin	Erythromycin	Kanamycin	Rifampicin	Tetracycline
HNL 1	<0.5	128	1	128	128	2
HNL 2	<0.5	128	1	128	128	2
HNL 4	<0.5	128	1	128	128	2
HNL 6	<0.5	128	1	128	128	2
HNL 7	<0.5	128	1	128	128	2
HNL 9	<0.5	128	1	128	128	2
HNL 10	<0.5	128	1	128	128	2

**Table 3:** Comparison of means of quantified short-chain fatty acids between isolates and between time points.

Short-chain fatty acid (mM)	Time (Day)	Isolates (HNL)								SEM	*p*-Value
		1	2	4	6	7	9	10	Control		
Formic acid	3	0.00	0.00 ^x^	0.00	0.00	0.00	0.00	0.00	0.00	0.000	-
	7	4.85 ^b^	7.33 ^ab,y^	7.59 ^ab^	9.44 ^a^	8.43 ^ab^	8.83 ^ab^	6.15 ^ab^	0.00 ^c^	1.026	<0.001
	14	6.98 ^b^	10.54 ^a,z^	10.34 ^a^	10.81 ^a^	8.05 ^ab^	7.17 ^b^	7.12 ^b^	0.00 ^c^	0.774	<0.001
	SEM	1.236	0.463	0.664	0.439	0.973	0.367	0.658	0.000		
Acetic acid	3	20.63 ^c,z^	22.26 ^c,z^	39.02 ^a,y^	38.55 ^a,y^	38.02 ^a,x^	26.18 ^b,z^	24.80 ^b,y^	22.4 ^c^	0.391	<0.001
	7	27.00 ^d,y^	35.40 ^c,y^	38.07 ^abc,y^	40.13 ^ab,y^	41.81 ^a,y^	36.06 ^c,y^	28.43 ^d,y^	21.88 ^e^	0.991	<0.001
	14	31.73 ^c,x^	41.55 ^b,x^	50.30 ^a,x^	50.99 ^a,x^	48.34 ^a,x^	40.84 ^b,x^	40.01 ^b,z^	24.59 ^d^	0.523	<0.001
	SEM	0.175	0.757	0.591	1.225	1.014	0.269	1.026	0.023		
Propionic acid	3	5.35 ^c,x^	6.13 ^c,x^	11.86 ^a,x^	10.87 ^b,x^	10.46 ^a,x^	5.72 ^c,x^	5.62 ^c,x^	5.4 ^c^	0.185	<0.001
	7	4.27 ^b,y^	4.67 ^ab,y^	4.14 ^b,y^	4.59 ^ab,y^	4.07 ^b,y^	4.19 ^b,y^	4.40 ^ab,y^	5.10 ^a^	0.160	0.044
	14	0.00 ^c,z^	0.00 ^c,z^	0.00 ^c,z^	0.00 ^c,z^	6.59 ^a,z^	0.00 ^c,z^	0.00 ^c,z^	4.6 ^b^	0.011	<0.001
	SEM	0.136	0.285	0.078	0.128	0.134	0.057	0.076	0.057		
Butyric acid	3	50.51 ^ab^	59.37 ^a^	74.56 ^a,z^	77.97 ^a^	77.70 ^a^	49.63 ^ab^	49.99 ^ab^	18.79 ^b^	6.327	0.025
	7	121.03 ^a^	121.44 ^a^	118.74 ^a,y^	120.09 ^a^	118.07 ^a^	117.74 ^a^	116.49 ^a^	15.1 8 ^b^	3.656	<0.001
	14	117.99 ^a^	120.84 ^a^	124.31 ^a,x^	123.21 ^a^	124.04 ^a^	119.75 ^a^	119.61 ^a^	14.5 7 ^b^	4.323	0.002
	SEM	0.796	3.247	0.920	1.866	4.691	1.000	1.146	0.459		

^a, b, c^ significant mean differences between isolates per time point. ^x, y, z^ mean difference among time points of each isolate with significant linear trend (*p* < 0.0001).

## References

[1] Kuhnert P., Brodard I., Alsaaod M., Steiner A., Stoffel M.H., Jores J. (2020). *Treponema phagedenis* (ex Noguchi 1912) Brumpt 1922 sp. nov., nom. rev., isolated from bovine digital dermatitis. Int. J. Syst. Evol. Microbiol..

[2] Yano T., Yamagami R., Misumi K., Kubota C., Kyaw K.M., Hayashi T., Yoshitani K., Ohtake O., Misawa N. (2009). Genetic heterogeneity among strains of *Treponema phagedenis*-like spirochetes isolated from dairy cattle with papillomatous digital dermatitis in Japan. J. Clin. Microbiol..

[3] Demirkan I., Walker R.L., Murray R.D., Blowey R.W., Carter S.D. (1999). Serological Evidence of Spirochaetal Infections Associated with Digital Dermatitis in Dairy Cattle. Vet. J..

[4] Evans N.J., Murray R.D., Carter S.D. (2016). Bovine digital dermatitis: Current concepts from laboratory to farm. Vet. J..

[5] Klitgaard K., Nielsen M.W., Ingerslev H.C., Boye M., Jensen T.K. (2014). Discovery of bovine digital dermatitis-associated *Treponema* spp. in the dairy herd environment by a targeted deep-sequencing approach. Appl. Environ. Microbiol..

[6] Cheli R., Mortellaro C. Digital dermatitis in cattle. Proceedings of the 8th International Meeting on Diseases of Cattle.

[7] Moreira T.F., Facury Filho E.J., Carvalho A.U., Strube M.L., Nielsen M.W., Klitgaard K., Jensen T.K. (2018). Pathology and bacteria related to digital dermatitis in dairy cattle in all year round grazing system in Brazil. PLoS ONE.

[8] Wilson-Welder J.H., Alt D.P., Nally J.E. (2015). Digital dermatitis in cattle: Current bacterial and immunological findings. Animals.

[9] Mamuad L.L., Joo B., Al S., Espiritu H.M., Jeong S., Kim W., Lee S., Cho Y. (2020). *Treponema* spp., the dominant pathogen in the lesion of bovine digital dermatitis and its characterization in dairy cattle. Vet. Microbiol..

[10] Zinicola M., Lima F., Lima S., Machado V., Gomez M. (2015). Altered Microbiomes in Bovine Digital Dermatitis Lesions, and the Gut as a Pathogen Reservoir. PLoS ONE.

[11] Sullivan L.E., Clegg S.R., Angell J.W., Newbrook K., Blowey R.W., Carter S.D., Bell J., Duncan J.S., Grove-White D.H., Murray R.D. (2015). High-level association of bovine digital dermatitis *Treponema* spp. with contagious ovine digital dermatitis lesions and presence of *Fusobacterium necrophorum* and *Dichelobacter nodosus*. J. Clin. Microbiol..

[12] Nielsen M.W., Strube M.L., Isbrand A., Al-Medrasi W.D.H.M., Boye M., Jensen T.K., Klitgaard K. (2016). Potential bacterial core species associated with digital dermatitis in cattle herds identified by molecular profiling of interdigital skin samples. Vet. Microbiol..

[13] Mushtaq M., Manzoor S., Pringle M., Rosander A., Bongcam-Rudloff E. (2015). Draft genome sequence of “*Treponema phagedenis*” strain V1, isolated from bovine digital dermatitis. Stand. Genomic Sci..

[14] Demirkan I., Erdoğan M., Demirkan A.Ç., Bozkurt F., Altındiş M., Navruz F.Z., Köse Z. (2018). Isolation and identification of *Treponema pedis* and *Treponema phagedenis*-like organisms from bovine digital dermatitis lesions found in dairy cattle in Turkey. J. Dairy Sci..

[15] Nally J.E., Hornsby R.L., Alt D.P., Whitelegge J.P. (2019). Phenotypic and proteomic characterization of treponemes associated with bovine digital dermatitis. Vet. Microbiol..

[16] Pringle M., Bergsten C., Fernström L.L., Höök H., Johansson K.E. (2008). Isolation and characterization of *Treponema phagedenis*-like spirochetes from digital dermatitis lesions in Swedish dairy cattle. Acta Vet. Scand..

[17] Trott D.J., Moeller M.R., Zuerner R.L., Goff J.P., Waters W.R., Alt D.P., Walker R.L., Wannemuehler M.J. (2003). Characterization of Treponema phagedenis-like spirochetes isolated from papillomatous digital dermatitis lesions in dairy cattle. J. Clin. Microbiol..

[18] Wilson-Welder J.H., Elliott M.K., Zuerner R.L., Bayles D.O., Alt D.P., Stanton T.B. (2013). Biochemical and molecular characterization of *Treponema phagedenis*-like spirochetes isolated from a bovine digital dermatitis lesion. BMC Microbiol..

[19] Demirkan I., Williams H.F., Dhawi A., Carter S.D., Winstanley C., Bruce K.D., Hart C.A. (2006). Characterization of a spirochaete isolated from a case of bovine digital dermatitis. J. Appl. Microbiol..

[20] Lee I., Kim Y.O., Park S.C., Chun J. (2016). OrthoANI: An improved algorithm and software for calculating average nucleotide identity. Int. J. Syst. Evol. Microbiol..

[21] Evans N.J., Brown J.M., Demirkan I., Birtles R., Hart C.A., Carter S.D. (2008). In vitro susceptibility of bovine digital dermatitis associated spirochaetes to antimicrobial agents. Vet. Microbiol..

[22] Yano T., Moe K.K., Chuma T., Misawa N. (2010). Antimicrobial susceptibility of *Treponema phagedenis*-like spirochetes isolated from dairy cattle with papillomatous digital dermatitis lesions in Japan. J. Vet. Med. Sci..

[23] Miguel M.A., Lee S.S., Mamuad L.L., Choi Y.J., Jeong C.D., Son A., Cho K.K., Kim E.T., Kim S.B., Lee S.S. (2019). Enhancing Butyrate Production, Ruminal Fermentation and Microbial Population through Supplementation with *Clostridium saccharobutylicum*. J. Microbiol. Biotechnol..

[24] Dworkin M., Falkow S., Rosenberg E., Schleifer K.-H., Stackebrandt E. (2006). Proteobacteria: Delta and Epsilon Subclasses, Deeply Rooting Bacteria. The Prokaryotes, A Handbook on the Biology of Bacteria.

[25] Matilla M.A., Krell T. (2018). The effect of bacterial chemotaxis on host infection and pathogenicity. FEMS Microbiol. Rev..

[26] Lux R., Miller J.N., Park N.H., Shi W. (2001). Motility and chemotaxis in tissue penetration of oral epithelial cell layers by *Treponema denticola*. Infect. Immun..

[27] Chu L., Kennell W., Holt S.C. (1994). Characterization of hemolysis and hemoxidation activities by *Treponema denticola*. Microb. Pathog..

[28] Scott D., Chan E.C.S., Siboo R. (1996). Iron acquisition by oral hemolytic spirochetes: Isolation of a hemin-binding protein and identification of iron reductase activity. Can. J. Microbiol..

[29] Margulis L., Maniotis A., MacAllister J., Scythes J., Brorson O., Hall J., Krumbein W.E., Chanman M.J. (2009). Spirochete round bodies syphilis, lyme disease & AIDS: Resurgence of “the great imitator”?. Symbiosis.

[30] Moore L.J., Woodward M.J., Grogono-Thomas R. (2005). The occurrence of treponemes in contagious ovine digital dermatitis and the characterisation of associated *Dichelobacter nodosus*. Vet. Microbiol..

[31] Demirkan I., Evans N.J., Singh P., Brown J.M., Getty B., Carter S.D., Timofte D., Hart C.A., Vink W.D., Birtles R.J. (2009). Association of Unique, Isolated Treponemes with Bovine Digital Dermatitis Lesions. J. Clin. Microbiol..

[32] Krieg N.R., Staley J.T., Brown D.R., Hedlund B.P., Paster B.J., Ward N.L., Ludwig W., Whitman W.B., Parte A.C. (2011). The Bacteroidetes, Spirochaetes, Tenericutes (Mollicutes), Acidobacteria, Fibrobacteres, Fusobacteria, Dictyoglomi, Gemmatimonadetes, Lentisphaerae, Verrucomicrobia, Chlamydiae, and Planctomycetes. Bergey’s Manual of Systematic Bacteriology.

[33] Van Horn K.G., Smibert R.M. (1981). Fatty acid requirement of *Treponema denticola* and *Treponema vincentii*. Can. J. Microbiol..

[34] O’Leary W. (1989). Practical Handbook of Microbiology.

